# Editorial: Nutrition for humanity in the Anthropocene - for healthier people on a healthier planet

**DOI:** 10.3389/fnut.2024.1467576

**Published:** 2024-08-23

**Authors:** Martin Kussmann, Matthew Hayek, Silvia Berciano

**Affiliations:** ^1^Kompetenzzentrum für Ernährung (KErn), Bayerische Landesanstalt für Ernährung, Landwirtschaft und Forsten (LfL), Freising, Germany; ^2^Kussmann Biotech GmbH, Nordkirchen, Germany; ^3^Department of Environmental Studies, New York University, New York, NY, United States; ^4^WISEcode, Reno, NV, United States; ^5^Friedman School of Nutrition Science and Policy, Tufts University, Boston, MA, United States

**Keywords:** nutrition, health, sustainability, humanity, Anthropocene, bioactive, nutrient

Currently, human consumption surpasses the planet's capacity to sustainably support health and maintain biodiverse ecological systems. The two major challenges to be met for preserving a healthy human life on a healthy planet are sustainable generation und use of energy and food. This Research Topic focuses on the critical need for a sustainable food system, a cornerstone in building a viable and healthy future for both humanity and the planet.

As we navigate the Anthropocene, humanity faces enormous environmental challenges. Our increasing population, which stands at 8 billion today and is projected to reach 10 billion by the end of this century, places significant stress on land, water and biodiversity and contributes to the acceleration of climate change. These issues call for an overhaul and upgrade of our food and healthcare system, to render both more resilient. Fortunately, we are armed with an unprecedented array of knowledge, cutting-edge technologies, and innovative tools. The converging revolutions in biotechnology and information science present substantial opportunities. Furthermore, the increasing international cooperation in research, economy, and policy-making provides an emerging foundation for addressing these global challenges at scale. Leveraging this collective expertise and collaborative spirit is essential in our pursuit of a sustainable future.

Nutrition occupies a pivotal role at the intersection of human health, animal welfare, and environmental sustainability. It encompasses the management of global food supplies to meet population demands sustainably, the advancement of personalized and precision nutrition to optimize individual health outcomes, and the exploration of bioactive compounds in natural food sources. The objectives of modern nutritional science are to support human health and wellbeing, contribute to disease prevention, and extend the health span concomitant with increasing life expectancies. Concurrently, nutrition must be practiced with an acute awareness of sustainable resource utilization, aiming to mitigate lasting detrimental effects on the environment and climate.

To meet these seemingly overwhelming and partly competing challenges of our era, nutrition science is progressing toward a systems-based, translational approach. This (r)evolution requires the development of sustainable food systems, a healthcare system that balances efficiency with affordability, and the creation of nutritional and dietary plans customized for diverse consumer and patient needs and demographics. Achieving a sustainable food system necessitates a greater reliance on plant-based sources for essential macro- and micronutrients as well as phytochemicals. An efficient and cost-effective healthcare system should integrate comprehensive nutritional strategies - encompassing general, medical, and clinical nutrition - to augment and complement the traditional pharmaceutical interventions. Personalized nutrition, moreover, depends on translational research that incorporates detailed phenotyping of participants to ensure the representation of various population segments.

Under this scope and context, we present in this Research Topic scientific work that addresses the interconnected streams of sustainable nutrition and healthcare, as well as the advancement of nutrition as a translational science. These contributions utilize systems biology and data science to elucidate intricate physiological processes, thereby reinforcing the conceptual framework of nutrition as a systems science. The Research Topic includes studies that utilize omics technologies, artificial intelligence, and bioinformatics to identify and validate plant-based sources for high-quality proteins, micronutrients, and bioactive compounds. Furthermore, the contributions emphasize the role of diet in maintaining health and preventing disease, particularly focusing on conditions that can be positively influenced by nutritional interventions. Additionally, this body of work highlights how integrating indigenous knowledge with contemporary scientific approaches can offer innovative solutions to current food system challenges.

Our Research Topic encompasses seven articles which we herewith introduce in more detail, zooming in from nutrition policies and concepts via healthy and sustainable diets to leveraging natural bioactives:

**Nutrition policy**: Rifkin outlines a framework for nutrition policy tailored to address the contemporary challenges posed by climate change and public health. In his article, Rifkin advocates for four essential components within this framework: evidence-based government nutrition guidelines, promotion of healthy eating patterns, ensuring nutrition security, and implementing effective nutrition education programs. Such a comprehensive policy approach could significantly enhance societal resilience in the face of environmental and health adversities.

**Dietary guidelines**: van Dooren et al. discuss the integration of environmental sustainability into food-based dietary guidelines. Historically focused on health, the scope of food-based dietary guidelines (FBDGs) has evolved to include environmental considerations, as recommended by the Food and Agriculture Organization (FAO), the World Health Organization (WHO), and the Federation of European Nutrition Societies (FENS). van Dooren et al. categorize current FBDG strategies that embrace environmental sustainability into four distinct approaches: supplemental advice and consumer guidance; synergy demonstrations; diet modeling; and the incorporation of sustainable development goals.

**Precision nutrition**: Berciano et al. review and discuss the current status and future prospects of Precision Nutrition, from a scientific, societal, and economic perspective. The process starts with individual data collection, including biological, dietary, and other lifestyle factors. Data integration and interpretation enable personalized recommendations, which in turn can induce positive behavioral change, ultimately resulting in improved health outcomes and thereby fostering a more prevention-centric, effective, and sustainable healthcare model.

**Healthy and sustainable diets**: Webb et al. provide insights into assessing diets that are not only healthy but also environmentally sustainable, affordable, and equitable. Various metrics, datasets, and analytical techniques have been deployed to assess the impact of dietary choices on greenhouse gas emissions, environmental degradation, food and nutrition security, and health outcomes. This work by Webb et al. represents a comprehensive approach to holistically understand multifaceted diet-outcome relationships.

**Nutrient efficiency**: Rezzi et al. extend the concept of “protein efficiency” to encompass all nutrients, advocating for “nutrient efficiency” as a key metric for nutritional health and sustainability. This approach measures the proportion of dietary nutrients that contribute to meeting dietary requirements and providing health benefits, thus bridging the gap between nutritional science and sustainability efforts.

**Natural bioactives**: Kussmann et al. show how to harness the potential of bioactive compounds found in nature for better human and planetary health. The authors discuss how advancements in omics and computational biology can be integrated into Precision Nutrition, merging the disciplines of nutrition and medicine to devise efficient and affordable healthcare solutions. However, translation requires bold changes in our food system, from agriculture through production and distribution to consumption. Investing in bioactive-based solutions is an opportunity to protect biodiversity and the health of our soils, waters, and the atmosphere, while creating value for consumers, patients, communities, and stakeholders.

**Indigenous nutrition knowledge**: Birch et al. report on the nutritional composition of Australian native grains used by First Nations people, examining the potential of pre-colonial food systems to address modern problems such as land degradation, reduced ecological resilience, and food insecurity. The authors advocate for the modern reintegration of these ancient food systems, ensuring they are applied with authenticity and integrity.

We hope that these articles can offer diverse and innovative perspectives to build food and healthcare systems that are more efficient, resilient, and equitable, outlining exemplary solutions to sustainably. Nourishing the world population while preserving our one and only earth. In the era of the Anthropocene.

